# Editorial: The Metabolism of the Neuron-Glia Unit

**DOI:** 10.3389/fncel.2021.791389

**Published:** 2021-11-05

**Authors:** Yannick Poitelon, Lance A. Johnson, Marie-Ève Tremblay

**Affiliations:** ^1^Department of Neuroscience and Experimental Therapeutics, Albany Medical College, Albany, NY, United States; ^2^Department of Physiology and Sanders Brown Center on Aging, University of Kentucky, Lexington, KY, United States; ^3^Division of Medical Sciences, University of Victoria, Victoria, BC, Canada

**Keywords:** metabolism, astrocyte, oligodendrocyte, microglia, Schwann cell, neuron, disease

Initially thought to simply hold the nervous system together, glial cells are now known to regulate a variety of critical functions in the nervous system. These functions include providing trophic support to neurons, ensuring fast impulse conduction, modulating synaptic formation, maturation, action, plasticity, and maintaining parenchymal homeostasis by actively regulating metabolites and clearing cell debris. Whilst the concept of metabolic coupling between neurons and glia has been entertained for years, especially for astrocytes, more recent work has resulted in major advances in our understanding of the molecular and cellular aspects of this coupling, notably investigating novel glial cell types, utilizing cutting edge technical tools, and developing unique animal models. The collection of articles in this Research Topic adds further insight to this growing knowledge base and provides new information worth considering in future research. Here, we present an overview about the included review articles as well as novel method and original research.

Throughout the nervous system tissues, neurons are closely associated with glial cells, including astrocytes, oligodendrocytes, microglia, and Schwann cells. Their dynamic interactions are important for nervous system development and function throughout adult life, as neurons rely on glial cells to fulfill their energetic demands. There is also emerging evidence that metabolic alterations in glial cells can modify their function and result in neuronal dysfunction, while every neurological disease involves some glial component, whether it be a primary or secondary cause for the disease.

In their review Afridi et al., examine recent developments that implicate the regulation of microglial and astrocytic phenotypic changes and their consequences on neuron–glia interactions in neurological disorders that include multiple sclerosis, Parkinson's disease, Alzheimer's disease, amyotrophic lateral sclerosis, and cerebral ischemia. They also discuss the challenges and possibilities of targeting glial metabolism as a strategy to treat neurological disorders. Shippy et al. discuss a similar theme in their review while focusing on Alzheimer's disease and the involvement of neuron-glia metabolic coupling in the context of neurodegeneration. The authors particularly discuss how microglial metabolism affects immune responses in dementia and how modulation of immunometabolism may be a valuable strategy for the development of advanced therapeutics to treat human diseases.

In their contribution, Niiyama et al. examine sex-dependent changes in spine structure of granule neurons of the dentate gyrus in male and female mice with hyperthyroidism, a condition which affects metabolism. The authors report that both sex-independent increases in spine density and sex-dependent alterations of spine morphology. These results may help to understand the functions of thyroid hormones in the central nervous system, and highlight the importance of fully understanding sex-dependent effects to better treat or prevent neurological symptoms related to thyroid dysfunction.

Jha et al. discuss the importance of monocarboxylate transporters (MCTs) by summarizing the emerging roles of lactate, pyruvate and ketone bodies transporters in glia-neuron metabolic interactions of the central and peripheral nervous systems. Though the metabolic support of neurons through the shuttling of lactate by glial cells was proposed many years ago, it is in part thanks to conditional knockout models targeting MCTs that the lactate shuttle hypothesis is now well-acknowledged. The authors provide a comprehensive analysis of the expression patterns and functions of MCTs and highlight their roles in modulating glia-neuron bidirectional metabolic crosstalk.

Boucanova et al. more deeply examine the role of Schwann cells, the principal glia in the peripheral nervous system of higher vertebrates, in the metabolic support of axons. Schwann cells are mainly known for wrapping and myelinating axons. However, the trophic support of axons by Schwann cell is beginning to be considered, as these glial cells have been shown to exchange both neurotransmitters and glycolysis by-products with axons. In this minireview, the authors present the role of Schwann cell as metabolic supporters of axonal health and make the case that characterization of the Schwann cell-axon metabolic relationship is necessary to better understand peripheral neuropathies.

Lana et al. describe how perturbation in the regulation of brain energy metabolism–not only in neurons, but also in astrocytes and microglia–may be one of the pathophysiological mechanisms of neurodegeneration, especially in hypoxia/ischemia. In this review, the authors analyze published data to demonstrate that glial responses to the same hypoxic insult are not equal, as the quantitative and morpho-functional alterations of the neuron–astrocyte–microglia response varied significantly in the hippocampus between the CA1 (more vulnerable to ischemia) and CA3 (less vulnerable to ischemia) regions.

Finally, Lerchundi et al. developed a new strategy to selectively permeabilize neurons and astrocytes in organotypic brain slice cultures to enable a free exchange of ATP across the plasma membrane. They show that their new procedure enables the quantitative measurement of changes in intracellular ATP, through ATeam, a FRET-based nanosensor. This novel methodology is of high interest for our Research Topic, as it is applicable to animals expressing ATeam in the brain and may be employed for the calibration of other genetically encoded biosensors sensitive to lactate, pyruvate, or glucose.

We thank all authors for their valuable contributions, and hope that the readers of this Research Topic will enjoy as much as we did the expert opinions and excellent original research presented.

## Author Contributions

YP wrote the first draft of the editorial. All the authors have contributed to this editorial, by editing and writing comments for the different articles.

## Funding

YP was supported by the National Institutes of Health (NS110627). LJ was supported by the National Institutes of Health (R01AG060056 and R01AG062550). M-ÈT was a Tier II Canada Research Chair in *Neurobiology of Aging and Cognition*.

## Conflict of Interest

The authors declare that the research was conducted in the absence of any commercial or financial relationships that could be construed as a potential conflict of interest.

## Publisher's Note

All claims expressed in this article are solely those of the authors and do not necessarily represent those of their affiliated organizations, or those of the publisher, the editors and the reviewers. Any product that may be evaluated in this article, or claim that may be made by its manufacturer, is not guaranteed or endorsed by the publisher.

